# Crystal structure and Hirshfeld surface analysis of 4-(naphthalen-2-yl)-*N*-[(*Z*)-4-propoxybenzyl­idene]-1,3-thia­zol-2-amine

**DOI:** 10.1107/S2056989020006611

**Published:** 2020-05-29

**Authors:** Ropak A. Sheakh Mohamad, Wali M. Hamad, Hashim J. Aziz

**Affiliations:** a Salahaddin University, College of Science, Department of Chemistry, Erbil, Iraq; b Koya University, Faculty of Science and Health, Department of Chemistry, Koya, Iraq; c Salahaddin University, College of Education, Department of Chemistry, Erbil, Iraq

**Keywords:** Crystal structure, heterocyclic compound, thia­zole, Schiff base, Hirshfeld surface analysis, crystal structure

## Abstract

The asymmetric unit of the title compound, C_23_H_20_N_2_OS, contains one slightly bent mol­ecule. The naphthalene ring system and the thia­zole ring are twisted with respect to each other, making a dihedral angle of 13.69 (10)°; the anisole ring is inclined to the plane of the naphthalene ring system, the dihedral angle being 14.22 (12)°. In the crystal structure, mol­ecules are linked by C—H⋯π inter­actions, resulting in the formation of sheets parallel to (100). Within the sheets, very weak π–π stacking inter­actions lead to additional stabilization. Hirshfeld surface analysis and fingerprint plots reveal that the cohesion in the crystal structure is dominated by H⋯H (42.5%) and C⋯H/H⋯C (37.2%) contacts.

## Abstract 2

In the crystal, the mol­ecules are linked by weak C—H⋯π hydrogen bonds and very weak π–π stacking inter­actions. Two-dimensional fingerprint plots show that the largest contributions to the crystal cohesion come from H⋯H and C⋯H/H⋯C inter­actions.

## Chemical context   

A Schiff base (Schiff, 1864 ▸) is a compound having the general formula *R*N=C*R*_2_ (*R* = H, hydro­carb­yl) and thus belongs to the family of imines (McNaught & Wilkinson, 1997 ▸). The chemistry of Schiff bases and their derivatives has been an inter­esting field of research since their discovery. Subsequently, Schiff bases have constituted a significant class of compounds for new drug development, exhibiting biological activities including anti­microbial, anti-tuberculosis, anti­oxidant, anti-inflammatory, anti­convulsant, anti­depressant, anxiolytic, anti­hypertensive, anti­cancer and anti­fungal properties. The search for Schiff base-containing compounds with more selective activity and lower side effects continues to be an active area in medicinal chemistry (Kumar *et al.*, 2017 ▸). Likewise, heterocyclic compounds play an essential role in medicinal chemistry, or as key templates for the development of various therapeutic agents. As part of this family, thia­zoles (Ghawla Amit *et al.*, 2014 ▸) and their derivatives have been found to possess anti­convulsant, anti­microbial, anti-inflammatory, anti­cancer, anti-HIV, anti­diabetic, anti-Alzheimer, anti­hypertensive, and anti­oxidant activities. As a result of their potent and significant biological activities, they have excellent pharmaceutical importance (Kaur & Goyal, 2018 ▸).
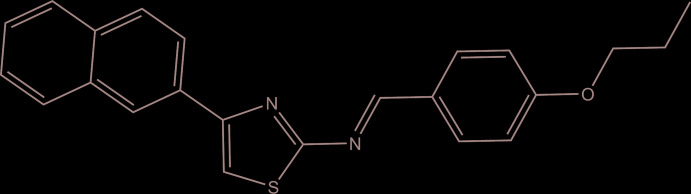


Here we report on the synthesis, structure determination and Hirshfeld analysis of a Schiff base, C_23_H_20_N_2_OS, (**I**), comprising a thia­zole entity.

## Structural commentary   

**Figure 1:**
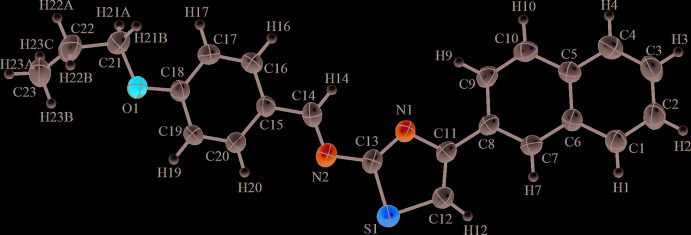
The mol­ecular structure of the title compound, with atom labelling. Displacement ellipsoids are drawn at the 40% probability level.

The asymmetric unit of (**I**) contains one mol­ecule (Fig. 1 ▸). The mol­ecule is slightly bent, with the naphthalene ring system and the thia­zole ring inclined to each other, subtending a dihedral angle of 13.69 (10)°; the anisole moiety is inclined to the plane of the naphthalene ring system, the dihedral angle being 14.22 (12)°. The C18—O1 and C21—O1 bond lengths are typical of single bonds (Table 1 ▸). The bond-length distribution in the thia­zole ring is normal. The C11—N1 bond has single-bond character and the C13—N1 bond double-bond character, with bond lengths of 1.380 (3) and 1.304 (3) Å, respectively.

## Supra­molecular features   

**Figure 2:**
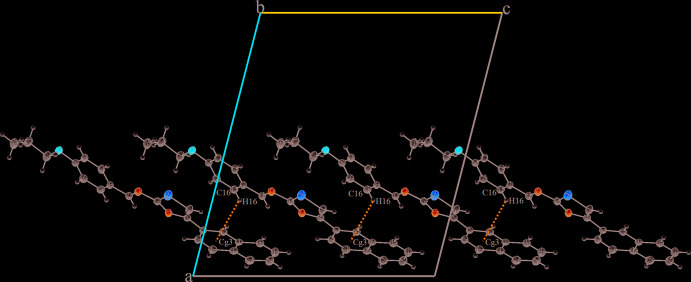
A view of the crystal packing of the title compound in a view along the *b* axis. C—H⋯π(ring) inter­actions are indicated by dashed lines.

**Figure 3:**
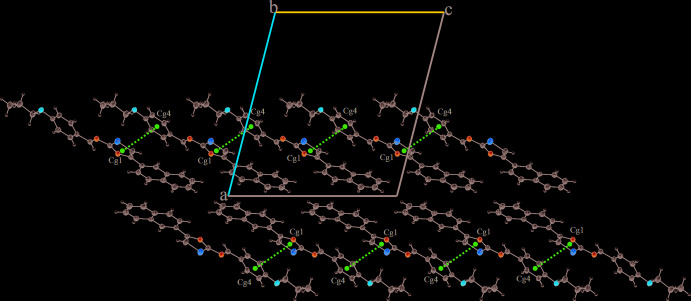
A view of the crystal packing of the title compound along the *b* axis. *π*(*Cg*1)⋯*π*(*Cg*4) inter­actions are indicated by dashed lines.

In the crystal structure, mol­ecules are connected into sheets extending along (100) by C4—H4⋯*Cg*3^i^ and C16—H16⋯*Cg*3^ii^ inter­actions (Table 2 ▸; Fig. 2 ▸), where *Cg*3 is the centroid of the C5–C10 ring. Within the sheets, very weak π–π stacking inter­actions are observed with a centroid-to-centroid distance *Cg*1⋯*Cg*4 = 4.494 (2) Å, where *Cg*1 and *Cg*4 are the centroids of the S1/C12/C11/N1/C13 and the C15–C20 phenyl ring, respectively (Fig. 3 ▸).

## Database survey   

A search of the Cambridge Structural Database (CSD, version 5.40, update November 2018; Groom *et al.*, 2016 ▸) for the 4-(4,6-dihydro­naphthalen-1-yl)thia­zol-2-amine moiety revealed two hits, *viz*. 4-(pyren-1-yl)-1,3-thia­zol-2-amine (pyrene thia­zole conjugate, PTC), C_19_H_12_N_2_S (SOPREW; Mahapatra *et al.*, 2014 ▸), and (*E*)-4-(4-chloro­phen­yl)-*N*-(1,3-benzodioxol-5-yl­methyl­ene)-5-(1*H*-1,2,4-tria­zol-1-yl)-1,3-thia­zol-2-amine, C_19_H_12_ClN_5_O_2_S (XAZJUE; Shao *et al.*, 2006 ▸). In the crystal packing of PTC, the two mol­ecules are connected into symmetrical dimers by pairs of N—H⋯N hydrogen bonds at a distance of 2.49 Å and are stacked along the *a* axis by weak aromatic π–π stacking inter­actions between the benzene rings in adjacent mol­ecules [centroid-to-centroid distances of 3.5741 (10) Å]. Distinctive bond lengths (*e.g*. N1—C11, N—C13, S1—C12, S1—C13) in (**I**) are the same within standard deviations as the corres­ponding bond lengths in the structure of XAZJUE. In XAZJUE, the mol­ecules are linked by weak C—H⋯O hydrogen bonds into a three-dimensional network.

## Hirshfeld surface analysis   

**Figure 4:**
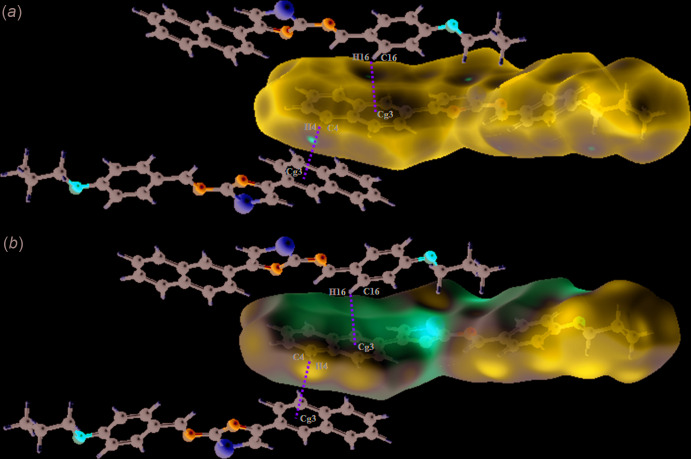
(*a*) Hirshfeld surfaces of the title compound mapped over *d_norm_*, with a fixed colour scale of −0.0638 (red) to 1.3242 (blue) a.u., and (*b*) the mol­ecular electrostatic potential surface of the title compound obtained over Hirshfeld surface containing C—H⋯π inter­actions, with a fixed colour scale of −0.049 (red) to 0.034 (blue) a.u..

Hirshfeld surface analysis (Spackman & Jayatilaka, 2009 ▸; McKinnon *et al.*, 2007 ▸) was carried out using *CrystalExplorer17.5* (Turner *et al.*, 2017 ▸). The Hirshfeld surface and their associated two-dimensional fingerprint plots were used to qu­antify the various inter­molecular inter­actions in (**I**). Hirshfeld surface analysis was performed using a standard (high) surface resolution with the three-dimensional *d*_norm_ surfaces mapped over a fixed colour scale of −0.0638 (red) to 1.3242 (blue) a.u., and the results are illustrated in Fig. 4 ▸ *a*. The red spots identified in Fig. 4 ▸ *a* correspond to the near-type H⋯π contacts resulting from hydrogen bonds of the type C—H⋯π(ring) (Table 2 ▸). The view of the three-dimensional Hirshfeld surface of the title compound plotted over electrostatic potentials with a fixed colour scale of −0.049 (red) to 0.034 (blue) a.u. is given in Fig. 4 ▸ *b*, emphasizing on C—H⋯π(ring) contacts.

**Figure 5:**
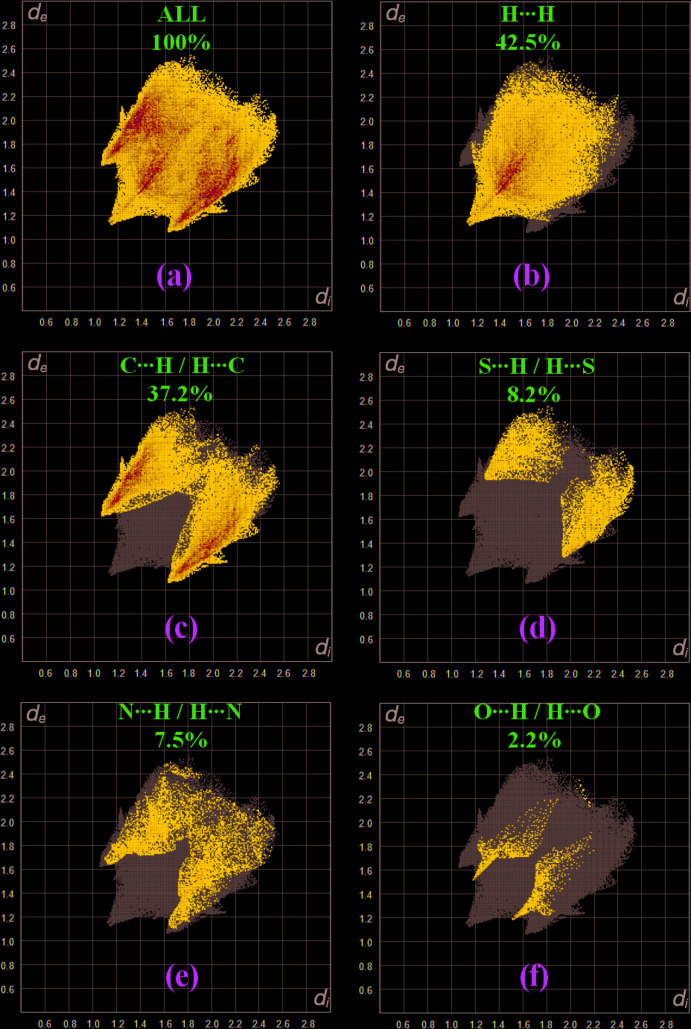
Two-dimensional fingerprint plots for the title compound, with a *d*_norm_ view (*a*), and delineated into relative contributions of the atom pairs to the Hirshfeld surface (*b*–*f*).

Fig. 5 ▸ *a* shows the two-dimensional fingerprint as the sum of all contacts contributing to the Hirshfeld surface indicated in normal mode. Fig. 5 ▸ *b* illustrates the two-dimensional fingerprint of (*d*_i_, *d*_e_) points related to H⋯H contacts that represent a 42.5% contribution in the title structure. In Fig. 5 ▸ *c*, two symmetrical wings on the left and right sides indicate C⋯H/H⋯C inter­actions with a contribution of 37.2%. Furthermore, there are S⋯H/H⋯S (8.2%; Fig. 5 ▸ *d*), N⋯H/H⋯N (7.5%; Fig. 5 ▸ *e*) and O⋯H/H⋯O (2.2%; Fig. 5 ▸ *f*) contacts contributing to the overall crystal packing of (**I**).

## Synthesis and crystallization   

Compound (**I**) was prepared by adding 4-*N*-propoxybenzaldehyde (0.145 g, 0.885 mmol) dropwise under constant stirring to a solution of 2-amino-4-(2-naphth­yl)thia­zole (0.2 g, 0.885 mmol) in 1-propanol (10 ml). The reaction was catalysed by NaOH (0.1 g), and the mixture stirred for 1 h in a water bath at approximately 278–283 K. The reaction was monitored by thin-layer chromatography (TLC) using ethyl acetate and *n*-hexane (3:7 *v*:*v*), and had an *R*_f_ of 0.675. The formed precipitate was filtered off, washed with 1-propanol, and dried. The resulting solid was further purified by washing with ethanol and diethyl ether. Single crystals of (**I**) for X-ray analysis were obtained by slow evaporation of an acetone solution (yield 60%, m.p. 411-413 K).

## Refinement   

Crystal data, data collection and structure refinement details are summarized in Table 3 ▸. The C-bound H atoms were placed in idealized positions and refined using a riding model with C—H = 0.93-0.97 Å and *U*_iso_(H) = 1.5*U*_eq_(C-meth­yl) or 1.2*U*_eq_(C) for other C-bound H atoms.

## Supplementary Material

Crystal structure: contains datablock(s) I. DOI: 10.1107/S2056989020006611/wm5556sup1.cif

Structure factors: contains datablock(s) I. DOI: 10.1107/S2056989020006611/wm5556Isup2.hkl

Supporting information file. DOI: 10.1107/S2056989020006611/wm5556Isup3.cml

CCDC reference: 2004309

Additional supporting information:  crystallographic information; 3D view; checkCIF report

## Figures and Tables

**Table 1:** Selected bond lengths (Å)

S1—C12	1.690 (3)	N1—C13	1.304 (3)
S1—C13	1.713 (3)	N1—C11	1.380 (3)
O1—C18	1.362 (3)	N2—C14	1.284 (3)
O1—C21	1.431 (3)	N2—C13	1.393 (3)

**Table 2:** Hydrogen-bond geometry (Å, °) *Cg*3 is the centroid of the C5–C10 ring.

*D*—H⋯*A*	*D*—H	H⋯*A*	*D*⋯*A*	*D*—H⋯*A*
C4—H4⋯*Cg*3^i^	0.93	2.83	3.496	130
C16—H16⋯*Cg*3^ii^	0.93	3.00	3.607	125

Symmetry codes: (i) 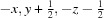; (ii) 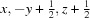.

**Table 3:** Experimental details

Crystal data	
Chemical formula	C_23_H_20_N_2_OS
*M*_r_	372.47
Crystal system, space group	Monoclinic, *P*2_1_/*c*
Temperature (K)	296
*a*, *b*, *c* (Å)	19.1636 (11), 6.0482 (3), 17.023 (1)
β (°)	104.370 (5)
*V* (Å^3^)	1911.32 (19)
*Z*	4
Radiation type	Mo *K*α
μ (mm^−1^)	0.18
Crystal size (mm)	0.68 × 0.29 × 0.05

Data collection	
Diffractometer	STOE *IPDS* 2
Absorption correction	Integration (*X-RED32*; Stoe & Cie, 2002 ▸)
*T*_min_, *T*_max_	0.919, 0.989
No. of measured, independent and observed [*I* > 2σ(*I*)] reflections	12341, 3770, 1951
*R*_int_	0.078
(sin θ/λ)_max_ (Å^−1^)	0.617

Refinement	
*R*[*F* ^2^ > 2σ(*F* ^2^)], *wR*(*F* ^2^), *S*	0.056, 0.087, 0.96
No. of reflections	3770
No. of parameters	245
H-atom treatment	H-atom parameters constrained
Δρ_max_, Δρ_min_ (e Å^−3^)	0.12, −0.16

Computer programs: *X-AREA* (Stoe & Cie, 2002 ▸), *X-RED* (Stoe & Cie, 2002 ▸), *SHELXT2017/1* (Sheldrick, 2015*a* ▸), *SHELXL2017/1* (Sheldrick, 2015*b* ▸), *PLATON* (Spek, 2020 ▸), *WinGX* (Farrugia, 2012 ▸).

## supplementary crystallographic information

### Crystal data

**Table:**

C_23_H_20_N_2_OS	*F*(000) = 784
*M**_r_* = 372.47	*D*_x_ = 1.294 Mg m^−^^3^
Monoclinic, *P*2_1_/*c*	Mo *K*α radiation, λ = 0.71073 Å
*a* = 19.1636 (11) Å	Cell parameters from 9076 reflections
*b* = 6.0482 (3) Å	θ = 1.8–31.6°
*c* = 17.023 (1) Å	µ = 0.18 mm^−^^1^
β = 104.370 (5)°	*T* = 296 K
*V* = 1911.32 (19) Å^3^	Stick, yellow
*Z* = 4	0.68 × 0.29 × 0.05 mm

### Data collection

**Table:**

STOE IPDS 2 diffractometer	3770 independent reflections
Radiation source: sealed X-ray tube, 12 x 0.4 mm long-fine focus	1951 reflections with *I* > 2σ(*I*)
Detector resolution: 6.67 pixels mm^-1^	*R*_int_ = 0.078
rotation method scans	θ_max_ = 26.0°, θ_min_ = 2.2°
Absorption correction: integration (X-RED32; Stoe & Cie, 2002)	*h* = −19→23
*T*_min_ = 0.919, *T*_max_ = 0.989	*k* = −7→7
12341 measured reflections	*l* = −20→20

### Refinement

**Table:**

Refinement on *F*^2^	0 restraints
Least-squares matrix: full	Hydrogen site location: inferred from neighbouring sites
*R*[*F*^2^ > 2σ(*F*^2^)] = 0.056	H-atom parameters constrained
*wR*(*F*^2^) = 0.087	*w* = 1/[σ^2^(*F*_o_^2^) + (0.0235*P*)^2^] where *P* = (*F*_o_^2^ + 2*F*_c_^2^)/3
*S* = 0.96	(Δ/σ)_max_ < 0.001
3770 reflections	Δρ_max_ = 0.12 e Å^−^^3^
245 parameters	Δρ_min_ = −0.16 e Å^−^^3^

### Special details

**Table:**

Geometry. All esds (except the esd in the dihedral angle between two l.s. planes) are estimated using the full covariance matrix. The cell esds are taken into account individually in the estimation of esds in distances, angles and torsion angles; correlations between esds in cell parameters are only used when they are defined by crystal symmetry. An approximate (isotropic) treatment of cell esds is used for estimating esds involving l.s. planes.

### Fractional atomic coordinates and isotropic or equivalent isotropic displacement parameters (Å^2^)

**Table:**

	*x*	*y*	*z*	*U*_iso_*/*U*_eq_	
S1	0.72771 (5)	0.01821 (13)	0.45111 (4)	0.0700 (2)	
O1	0.57359 (10)	0.7728 (3)	−0.00225 (10)	0.0614 (5)	
N1	0.78855 (12)	0.3982 (3)	0.46984 (12)	0.0518 (6)	
N2	0.71496 (12)	0.3175 (4)	0.33523 (12)	0.0574 (6)	
C15	0.69256 (14)	0.5693 (4)	0.22324 (14)	0.0471 (6)	
C6	0.88892 (13)	0.4646 (4)	0.76167 (14)	0.0486 (6)	
C8	0.84752 (13)	0.4163 (4)	0.61580 (15)	0.0467 (6)	
C11	0.80536 (13)	0.2954 (4)	0.54458 (15)	0.0481 (6)	
C7	0.85390 (13)	0.3407 (4)	0.69324 (15)	0.0497 (7)	
H7	0.834523	0.203683	0.700743	0.060*	
C20	0.63762 (14)	0.4417 (4)	0.17593 (14)	0.0552 (7)	
H20	0.626787	0.304989	0.194986	0.066*	
C18	0.61554 (13)	0.7170 (4)	0.07205 (14)	0.0494 (7)	
C13	0.74782 (15)	0.2697 (4)	0.41597 (15)	0.0526 (7)	
C5	0.91907 (14)	0.6724 (4)	0.75068 (16)	0.0520 (7)	
C9	0.87997 (14)	0.6206 (4)	0.60570 (16)	0.0542 (7)	
H9	0.877676	0.671926	0.553618	0.065*	
C19	0.59913 (14)	0.5150 (5)	0.10142 (14)	0.0557 (7)	
H19	0.562053	0.428985	0.070646	0.067*	
C10	0.91444 (14)	0.7434 (5)	0.67047 (16)	0.0581 (7)	
H10	0.935420	0.876963	0.661879	0.070*	
C17	0.67016 (15)	0.8446 (5)	0.11727 (16)	0.0570 (7)	
H17	0.681829	0.979223	0.097421	0.068*	
C14	0.72982 (13)	0.5003 (5)	0.30469 (13)	0.0548 (7)	
H14	0.765398	0.591233	0.335507	0.066*	
C16	0.70766 (14)	0.7699 (5)	0.19294 (15)	0.0574 (7)	
H16	0.744007	0.857655	0.224102	0.069*	
C1	0.89269 (14)	0.3919 (5)	0.84200 (16)	0.0592 (7)	
H1	0.873907	0.254632	0.850405	0.071*	
C12	0.77718 (16)	0.0891 (4)	0.54435 (16)	0.0617 (7)	
H12	0.784240	−0.001549	0.589769	0.074*	
C2	0.92348 (16)	0.5211 (6)	0.90667 (17)	0.0732 (9)	
H2	0.924833	0.472661	0.958877	0.088*	
C21	0.59299 (17)	0.9645 (5)	−0.04130 (15)	0.0699 (8)	
H21A	0.642670	0.953582	−0.044645	0.084*	
H21B	0.587991	1.096221	−0.010673	0.084*	
C4	0.95124 (16)	0.7990 (5)	0.81968 (18)	0.0696 (8)	
H4	0.971585	0.935292	0.813145	0.084*	
C3	0.95304 (17)	0.7257 (6)	0.89530 (18)	0.0761 (9)	
H3	0.974148	0.812465	0.939997	0.091*	
C22	0.54367 (17)	0.9770 (6)	−0.12479 (17)	0.0846 (10)	
H22A	0.554784	1.109652	−0.151339	0.102*	
H22B	0.494394	0.989504	−0.120138	0.102*	
C23	0.54931 (19)	0.7806 (7)	−0.17675 (18)	0.0990 (12)	
H23A	0.518100	0.801144	−0.229840	0.149*	
H23B	0.535317	0.649628	−0.152709	0.149*	
H23C	0.598123	0.765472	−0.180940	0.149*	

### Atomic displacement parameters (Å^2^)

**Table:**

	*U*^11^	*U*^22^	*U*^33^	*U*^12^	*U*^13^	*U*^23^
S1	0.0868 (6)	0.0596 (5)	0.0589 (4)	−0.0121 (5)	0.0092 (4)	0.0028 (4)
O1	0.0618 (12)	0.0702 (13)	0.0472 (10)	−0.0091 (10)	0.0041 (9)	0.0089 (9)
N1	0.0573 (14)	0.0520 (13)	0.0459 (13)	0.0032 (12)	0.0128 (11)	0.0038 (11)
N2	0.0621 (15)	0.0622 (15)	0.0476 (13)	0.0043 (13)	0.0130 (11)	0.0051 (12)
C15	0.0488 (16)	0.0527 (17)	0.0402 (13)	−0.0014 (14)	0.0117 (12)	−0.0031 (12)
C6	0.0387 (14)	0.0577 (17)	0.0493 (14)	0.0052 (14)	0.0107 (12)	0.0021 (14)
C8	0.0437 (15)	0.0471 (15)	0.0496 (16)	0.0062 (13)	0.0123 (12)	0.0080 (12)
C11	0.0460 (15)	0.0497 (16)	0.0494 (15)	0.0075 (13)	0.0134 (13)	0.0084 (13)
C7	0.0462 (16)	0.0480 (16)	0.0553 (16)	−0.0014 (13)	0.0136 (13)	0.0053 (13)
C20	0.0679 (19)	0.0524 (17)	0.0463 (15)	−0.0097 (15)	0.0159 (14)	0.0010 (13)
C18	0.0457 (15)	0.0578 (18)	0.0437 (15)	−0.0019 (14)	0.0093 (12)	−0.0005 (13)
C13	0.0562 (17)	0.0571 (18)	0.0452 (15)	0.0088 (15)	0.0140 (13)	0.0041 (14)
C5	0.0432 (16)	0.0509 (17)	0.0607 (18)	0.0018 (14)	0.0103 (13)	0.0005 (14)
C9	0.0556 (17)	0.0562 (17)	0.0497 (16)	0.0031 (15)	0.0107 (14)	0.0123 (13)
C19	0.0610 (17)	0.0587 (18)	0.0450 (14)	−0.0186 (16)	0.0085 (13)	−0.0065 (14)
C10	0.0540 (17)	0.0513 (17)	0.0680 (19)	0.0011 (15)	0.0135 (15)	0.0109 (15)
C17	0.0616 (18)	0.0525 (17)	0.0563 (17)	−0.0101 (15)	0.0135 (14)	0.0033 (13)
C14	0.0522 (17)	0.0664 (19)	0.0446 (14)	0.0054 (16)	0.0101 (12)	−0.0054 (15)
C16	0.0545 (17)	0.0619 (19)	0.0534 (17)	−0.0124 (15)	0.0085 (14)	−0.0033 (14)
C1	0.0509 (17)	0.073 (2)	0.0528 (17)	−0.0034 (15)	0.0119 (14)	0.0007 (15)
C12	0.0750 (19)	0.0530 (18)	0.0530 (16)	−0.0017 (17)	0.0079 (14)	0.0076 (14)
C2	0.072 (2)	0.094 (2)	0.0542 (17)	−0.005 (2)	0.0168 (15)	−0.0041 (18)
C21	0.085 (2)	0.0626 (19)	0.0587 (17)	−0.0039 (18)	0.0113 (16)	0.0097 (16)
C4	0.066 (2)	0.067 (2)	0.075 (2)	−0.0083 (17)	0.0159 (17)	−0.0091 (17)
C3	0.076 (2)	0.087 (3)	0.062 (2)	−0.012 (2)	0.0097 (17)	−0.0188 (18)
C22	0.084 (2)	0.099 (3)	0.0621 (19)	−0.002 (2)	0.0013 (17)	0.028 (2)
C23	0.100 (3)	0.139 (3)	0.0546 (19)	−0.035 (3)	0.0144 (19)	−0.005 (2)

### Geometric parameters (Å, º)

**Table:**

S1—C12	1.690 (3)	C9—C10	1.357 (4)
S1—C13	1.713 (3)	C9—H9	0.9300
O1—C18	1.362 (3)	C19—H19	0.9300
O1—C21	1.431 (3)	C10—H10	0.9300
N1—C13	1.304 (3)	C17—C16	1.386 (3)
N1—C11	1.380 (3)	C17—H17	0.9300
N2—C14	1.284 (3)	C14—H14	0.9300
N2—C13	1.393 (3)	C16—H16	0.9300
C15—C16	1.377 (3)	C1—C2	1.360 (4)
C15—C20	1.390 (3)	C1—H1	0.9300
C15—C14	1.454 (3)	C12—H12	0.9300
C6—C7	1.407 (3)	C2—C3	1.394 (4)
C6—C5	1.415 (3)	C2—H2	0.9300
C6—C1	1.421 (3)	C21—C22	1.501 (3)
C8—C7	1.372 (3)	C21—H21A	0.9700
C8—C9	1.413 (3)	C21—H21B	0.9700
C8—C11	1.473 (3)	C4—C3	1.354 (4)
C11—C12	1.359 (3)	C4—H4	0.9300
C7—H7	0.9300	C3—H3	0.9300
C20—C19	1.373 (3)	C22—C23	1.501 (4)
C20—H20	0.9300	C22—H22A	0.9700
C18—C17	1.372 (3)	C22—H22B	0.9700
C18—C19	1.386 (3)	C23—H23A	0.9600
C5—C4	1.409 (4)	C23—H23B	0.9600
C5—C10	1.413 (3)	C23—H23C	0.9600
			
C12—S1—C13	88.87 (14)	C18—C17—H17	120.5
C18—O1—C21	118.1 (2)	C16—C17—H17	120.5
C13—N1—C11	110.0 (2)	N2—C14—C15	121.8 (3)
C14—N2—C13	119.1 (2)	N2—C14—H14	119.1
C16—C15—C20	118.1 (2)	C15—C14—H14	119.1
C16—C15—C14	120.7 (3)	C15—C16—C17	121.8 (3)
C20—C15—C14	121.0 (2)	C15—C16—H16	119.1
C7—C6—C5	119.3 (2)	C17—C16—H16	119.1
C7—C6—C1	122.1 (3)	C2—C1—C6	120.6 (3)
C5—C6—C1	118.5 (2)	C2—C1—H1	119.7
C7—C8—C9	118.1 (2)	C6—C1—H1	119.7
C7—C8—C11	121.7 (2)	C11—C12—S1	111.4 (2)
C9—C8—C11	120.1 (2)	C11—C12—H12	124.3
C12—C11—N1	114.2 (2)	S1—C12—H12	124.3
C12—C11—C8	126.4 (2)	C1—C2—C3	120.5 (3)
N1—C11—C8	119.3 (2)	C1—C2—H2	119.8
C8—C7—C6	121.9 (2)	C3—C2—H2	119.8
C8—C7—H7	119.0	O1—C21—C22	107.8 (2)
C6—C7—H7	119.0	O1—C21—H21A	110.1
C19—C20—C15	120.8 (2)	C22—C21—H21A	110.1
C19—C20—H20	119.6	O1—C21—H21B	110.1
C15—C20—H20	119.6	C22—C21—H21B	110.1
O1—C18—C17	125.0 (2)	H21A—C21—H21B	108.5
O1—C18—C19	114.8 (2)	C3—C4—C5	121.1 (3)
C17—C18—C19	120.2 (2)	C3—C4—H4	119.4
N1—C13—N2	128.0 (2)	C5—C4—H4	119.4
N1—C13—S1	115.54 (19)	C4—C3—C2	120.5 (3)
N2—C13—S1	116.3 (2)	C4—C3—H3	119.8
C4—C5—C10	123.4 (3)	C2—C3—H3	119.8
C4—C5—C6	118.8 (2)	C21—C22—C23	113.4 (3)
C10—C5—C6	117.9 (2)	C21—C22—H22A	108.9
C10—C9—C8	121.3 (2)	C23—C22—H22A	108.9
C10—C9—H9	119.3	C21—C22—H22B	108.9
C8—C9—H9	119.3	C23—C22—H22B	108.9
C20—C19—C18	120.0 (2)	H22A—C22—H22B	107.7
C20—C19—H19	120.0	C22—C23—H23A	109.5
C18—C19—H19	120.0	C22—C23—H23B	109.5
C9—C10—C5	121.3 (3)	H23A—C23—H23B	109.5
C9—C10—H10	119.3	C22—C23—H23C	109.5
C5—C10—H10	119.3	H23A—C23—H23C	109.5
C18—C17—C16	119.0 (2)	H23B—C23—H23C	109.5
			
C13—N1—C11—C12	0.8 (3)	C15—C20—C19—C18	0.9 (4)
C13—N1—C11—C8	−176.4 (2)	O1—C18—C19—C20	−179.7 (2)
C7—C8—C11—C12	−9.7 (4)	C17—C18—C19—C20	−0.1 (4)
C9—C8—C11—C12	173.2 (3)	C8—C9—C10—C5	−0.2 (4)
C7—C8—C11—N1	167.1 (2)	C4—C5—C10—C9	−176.9 (3)
C9—C8—C11—N1	−10.0 (3)	C6—C5—C10—C9	2.3 (4)
C9—C8—C7—C6	2.6 (4)	O1—C18—C17—C16	178.6 (2)
C11—C8—C7—C6	−174.5 (2)	C19—C18—C17—C16	−1.0 (4)
C5—C6—C7—C8	−0.5 (4)	C13—N2—C14—C15	−174.3 (2)
C1—C6—C7—C8	176.9 (2)	C16—C15—C14—N2	177.6 (3)
C16—C15—C20—C19	−0.6 (4)	C20—C15—C14—N2	1.9 (4)
C14—C15—C20—C19	175.3 (2)	C20—C15—C16—C17	−0.5 (4)
C21—O1—C18—C17	8.3 (4)	C14—C15—C16—C17	−176.4 (2)
C21—O1—C18—C19	−172.1 (2)	C18—C17—C16—C15	1.3 (4)
C11—N1—C13—N2	174.8 (2)	C7—C6—C1—C2	−176.4 (3)
C11—N1—C13—S1	−0.4 (3)	C5—C6—C1—C2	1.1 (4)
C14—N2—C13—N1	7.1 (4)	N1—C11—C12—S1	−0.9 (3)
C14—N2—C13—S1	−177.7 (2)	C8—C11—C12—S1	176.1 (2)
C12—S1—C13—N1	0.0 (2)	C13—S1—C12—C11	0.5 (2)
C12—S1—C13—N2	−175.9 (2)	C6—C1—C2—C3	−1.1 (4)
C7—C6—C5—C4	177.3 (2)	C18—O1—C21—C22	174.3 (2)
C1—C6—C5—C4	−0.3 (4)	C10—C5—C4—C3	178.6 (3)
C7—C6—C5—C10	−1.9 (4)	C6—C5—C4—C3	−0.5 (4)
C1—C6—C5—C10	−179.5 (2)	C5—C4—C3—C2	0.6 (5)
C7—C8—C9—C10	−2.3 (4)	C1—C2—C3—C4	0.3 (5)
C11—C8—C9—C10	174.9 (2)	O1—C21—C22—C23	−61.0 (3)

### Hydrogen-bond geometry (Å, º)

*Cg*3 is the centroid of the C5–C10 ring.

**Table:**

*D*—H···*A*	*D*—H	H···*A*	*D*···*A*	*D*—H···*A*
C4—H4···*Cg*3^i^	0.93	2.83	3.496	130
C16—H16···*Cg*3^ii^	0.93	3.00	3.607	125

Symmetry codes: (i) −*x*, *y*+1/2, −*z*−1/2; (ii) *x*, −*y*+1/2, *z*+1/2.
